# Circadian Clock Dysfunction and Psychiatric Disease: Could Fruit Flies have a Say?

**DOI:** 10.3389/fneur.2015.00080

**Published:** 2015-04-20

**Authors:** Mauro Agostino Zordan, Federica Sandrelli

**Affiliations:** ^1^Department of Biology, University of Padova, Padova, Italy; ^2^Cognitive Neuroscience Center, University of Padova, Padova, Italy

**Keywords:** circadian clock, neuropsychiatric diseases, *Drosophila melanogaster*, gene X environment interactions, sleep, cognitive impairments, social interactions, behavioral traits

## Abstract

There is evidence of a link between the circadian system and psychiatric diseases. Studies in humans and mammals suggest that environmental and/or genetic disruption of the circadian system leads to an increased liability to psychiatric disease. Disruption of clock genes and/or the clock network might be related to the etiology of these pathologies; also, some genes, known for their circadian clock functions, might be associated to mental illnesses through clock-independent pleiotropy. Here, we examine the features which we believe make *Drosophila melanogaster* a model apt to study the role of the circadian clock in psychiatric disease. Despite differences in the organization of the clock system, the molecular architecture of the *Drosophila* and mammalian circadian oscillators are comparable and many components are evolutionarily related. In addition, *Drosophila* has a rather complex nervous system, which shares much at the cell and neurobiological level with humans, i.e., a tripartite brain, the main neurotransmitter systems, and behavioral traits: circadian behavior, learning and memory, motivation, addiction, social behavior. There is evidence that the *Drosophila* brain shares some homologies with the vertebrate cerebellum, basal ganglia, and hypothalamus-pituitary-adrenal axis, the dysfunctions of which have been tied to mental illness. We discuss *Drosophila* in comparison to mammals with reference to the: organization of the brain and neurotransmitter systems; architecture of the circadian clock; clock-controlled behaviors. We sum up current knowledge on behavioral endophenotypes, which are amenable to modeling in flies, such as defects involving sleep, cognition, or social interactions, and discuss the relationship of the circadian system to these traits. Finally, we consider if *Drosophila* could be a valuable asset to understand the relationship between circadian clock malfunction and psychiatric disease.

## Introduction

Mental health diseases (i.e., depressive syndromes, bipolar disorders, and schizophrenia) make up about 20% of all illnesses and approximately one person in four is afflicted by one form or other of this kind of disease during their lifetime. It is widely accepted that these disorders are complex pathologies, influenced by the interplay between several genes and multiple environmental factors (1, 2). Different lines of evidence suggest a link between the endogenous circadian system and at least some forms of these diseases (3–5).

**Figure 1:**
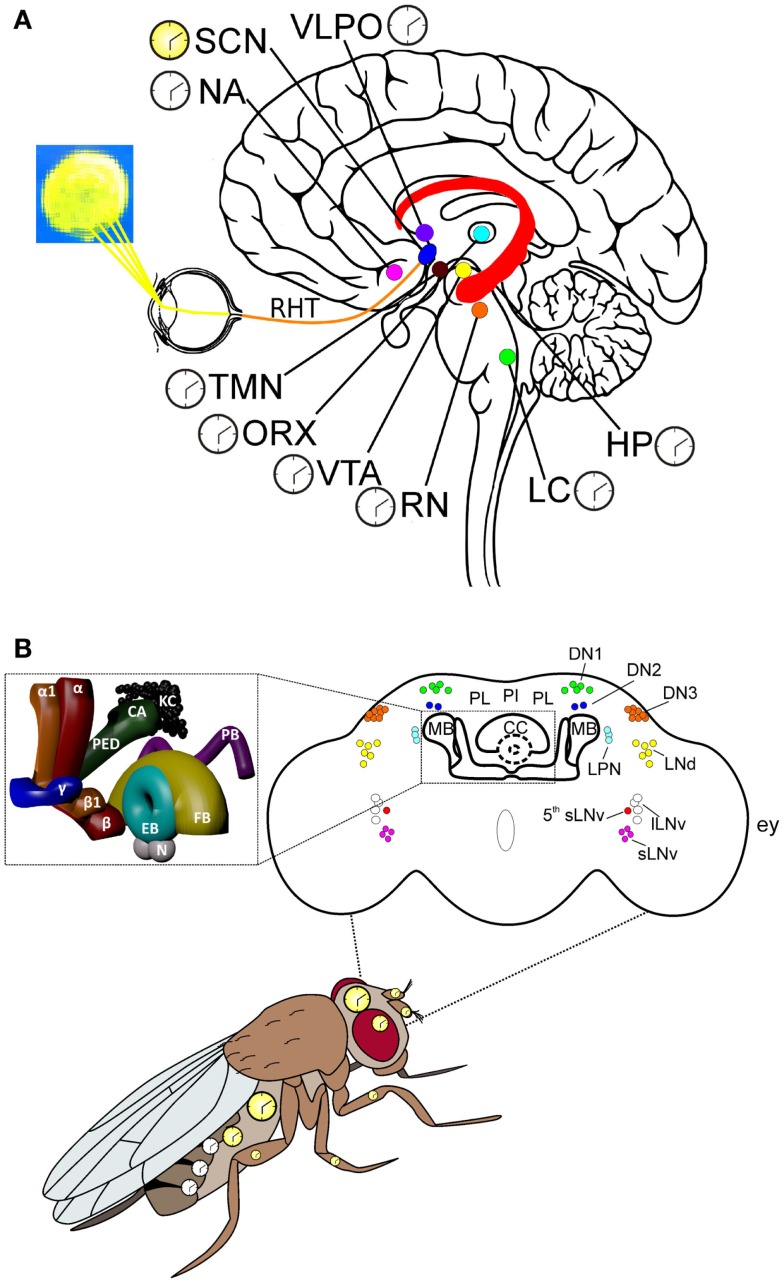
**Key centers of the mammalian and *Drosophila* brains and the circadian system in *Drosophila*. (A)** Some of the key areas of the human brain involved in the control of arousal/sleep, circadian rhythms, and cognitive processes related to motivation, emotions, and learning/memory. *Hippocampus (HP)*: this relatively large structure is part of the limbic system and is involved in stabilizing information in the consolidation of short-term to long-term memory. The HP is also implicated in spatial navigation. *Locus coeruleus (LC)*: norepinephrine from the LC mediates arousal, and primes the brain’s neurons to be activated by stimuli, and is involved with physiological responses to stress and panic. *Nucleus accumbens (NA)*: this region of the brain is involved in the cognitive elaboration of associative learning, motivation, pleasure, and addiction/reward. *Orexinergic neurons (ORX)*: these neurons produce the neurotransmitter orexin/hypocretin, which is involved in regulating arousal, wakefulness, and appetite. *Suprachiasmatic nucleus (SCN)*: these neurons are situated directly above the optic chiasm and are the seat of the central clock (yellow), which controls circadian rhythms through the action of different peptides and neurotransmitters on many other regions of the brain, which contain subsidiary clocks (white). *Raphe nuclei (RN)*: these serotoninergic nuclei are involved in a reciprocal feedback loop with the SCN to which they send information regarding levels of alertness; the SCN in turn sends connections to the RN; thus, influencing serotonin levels, which are involved in regulating sleep/wake states. *Ventrotegmental area (VTA)*: the dopaminergic neurons of this area of the brain are involved in cognitive processes related to addiction/reward and motivation. *Tuberomammillary nuclei (TMN)*: these histaminergic nuclei are involved in the control of arousal, learning, memory, sleep, and energy balance. *Retinohypothalamic tract (RHT)*: the retinohypothalamic tract originates in the intrinsically photosensitive retinal ganglion cells, which contain the photopigment melanopsin. The RHT axons, through the optic nerve and the optic chiasm, project to the suprachiasmatic nuclei. *Ventrolateral preoptic nucleus (VLPO)*: the VLPO is active during sleep and releases mainly GABA and galanin, which inhibit neurons that are involved in wakefulness and arousal (i.e., ORX, RN, LC, TMN). The latter groups of neurons are involved in a reciprocal feedback loop with the VLPO, thus contributing to the regulation of sleep. **(B)** The circadian system organization in *Drosophila*, with multiple oscillators located in the brain and body. The clocks in the brain, and in most of the peripheral tissues, are autonomous (yellow), while those located in the oenocytes resemble the mammalian subsidiary clocks (white), whose phase is controlled by the central brain clock; see text for details. Upper part: schematic representation of the adult fly brain, in which the relative positions of the circadian neurons, the mushroom bodies (MBs), the central complex (CC), the pars lateralis (PL), and the pars intercelebralis (PI) are reported [modified from Ref. (8, 9)]. The inset shows a 3D-reconstruction of the MBs and CC. KC: kenyon cells; CA: calyx; PED: pedunculus; α: α lobe; α1: α′ lobe; β: β lobe; β1: β′ lobe; γ: γ lobe; EB: ellipsoid body; FB: fan-shaped body; N: noduli; PB: protocerebral bridge; lLNv: large ventral lateral neurons; sLNv: small LNvs; 5th sLNv: the 5th PDF-negative sLNv; LPN: lateral posterior neuron; LNd: dorsal LNs; DN1: dorsal neurons group 1; DN2: DN group 2; DN3: DN group 3; ey: relative position of the compound eye respect to the brain.

In mammals, the circadian system is physiologically composed by an hierarchical network of clocks in which time-keeping molecular and cellular processes are integrated at the organismic level. The central circadian clock maps to the suprachiasmatic nucleus (SCN) of the hypothalamus, harboring an autonomous oscillator which is responsible for the synchronization with the daily environmental variations, such as the light: dark (LD) cycle [reviewed in Ref. (6, 7)] (Figure 1A). The SCN sends direct and indirect signals to all the peripheral clocks located in the brain and body, coordinating their rhythm and phase, so that they oscillate in phase with the SCN itself, with each other, and with the environment. Furthermore, the organism’s circadian clock-controlled phenotypes, such as the sleep/wake cycle, body temperature, and metabolism, are synchronized with the 24 h environmental variations.

In humans, clinical observations have shown that many psychiatric patients display abnormalities in circadian parameters such as cycles in body temperature, melatonin levels, blood pressure, cortisol secretion, and sleep/wake cycles (4, 10, 11). Several single nucleotide polymorphisms at the level of genes involved in the control of the circadian clock have been associated to different forms of psychiatric disorders (4, 12, 13). In addition, a post-mortem transcriptome analysis performed in individuals affected by depressive disorders showed that in different brain regions several circadian clock genes oscillate with a lower amplitude with respect to controls (14). On the other hand, in otherwise asymptomatic subjects, environmental conditions such as shift-work, chronic jet-lag, or “social” jet-lag, which are responsible for a misalignment between the environment and the internal circadian system, might represent risk factors for deterioration of mental health, cognition, and mood (15–17). Studies in mammalian models have shown that some genes such as m*Period2*, m*Clock*, and m*Rev-erb* α, which are fundamental elements of the molecular clockwork (see below), are also involved in the control of behaviors, which are considered psychiatric-like hallmarks in the modeling of mental illnesses in non-primate animals (18–22). Recent analyses have linked mCLOCK and mPER2 activities to the circadian regulation of dopamine synthesis (mCLOCK) and catabolism (mPER2) in brain regions considered fundamental in the control of psychiatric-like behaviors (18–21, 23), while REV-ERB α (22) has been demonstrated to be involved in the control of the hippocampal adult neurogenesis. Overall, these data have given rise to the hypothesis that an environmental and/or a genetically induced disruption of the circadian system might be associated with an increased susceptibility to psychiatric disease. This dysregulation of the circadian system might affect brain neuronal pathways and networks, i.e., through alterations in: monoamine neurotransmitter modulation, regulation of the hypothalamus-pituitary-adrenal axis (HPA), and neurogenesis, which are considered to be among the possible causes of these complex pathologies [reviewed in Ref. (24)]. However, the complexity of the circadian system organization in mammals, with the SCN coordinating all the other brain peripheral clocks, makes it difficult to determine whether the master clock exerts a direct influence on these neurological phenomena. In addition, some molecular components, known mainly for their involvement in circadian clock functions, might be associated to psychiatric illnesses through pleiotropic effects (i.e., additional clock-independent functions) (25).

Here, we speculate whether a relatively simple model organism, such as the fruit fly *Drosophila melanogaster*, might be informative in the study of the possible relationship between the circadian clock and human psychiatric disease. The *Drosophila* circadian clock is one of the best characterized at the molecular, physiological, and behavioral levels. Notwithstanding significant differences in the organization of the multiple-clock system at the organismic level, the overall molecular architecture of the *Drosophila* and mammalian circadian oscillators are comparable and many components are evolutionarily conserved. Recently, *Drosophila* has been proposed as a model organism for the study of psychiatric illnesses. Significantly, current research work is contributing to the definition of the details of the full set of a fly’s behavior, which includes motivation, social behavior, as well as some aspects of addiction, which are likely relevant features of neuropsychiatric disorders. In this review, we will describe the *Drosophila* characteristics regarding: (i) the organization of the brain and neurotransmitter systems, (ii) the architecture of the circadian clock, (iii) the clock-controlled behaviors, mainly in comparison with mammals. We will also sum up current knowledge relative to behavioral endophenotypes, which are amenable to modeling in flies, such as defects involving sleep, cognition, or social interactions, and discuss the relationship of the circadian system to these traits. Finally, we will speculate on the possible strategies based on the use of *Drosophila* as model organism to understand the relationship between circadian clock dysregulation and psychiatric disease.

## The *Drosophila melanogaster* Brain

From an evolutionary and phylogenetic standpoint, the invertebrate *D*. *melanogaster* is a member of the protostomes whereas vertebrates are deuterostomes; both of which are related by their belonging to the *Bilateria* (animals showing bilateral symmetry). The relevance of this distinction lies in the hypothesis, originally expressed by Anton Dohrn in 1875, that the vertebrate nervous system looks essentially like a dorsoventrally inverted version of the invertebrate nervous system. This idea has found solid support at the cellular and molecular levels (26). Furthermore, the dorsoventral and anteroposterior patterning of protostomes and deuterostomes show deep homology (i.e., phylogenetic conservation of genetic regulatory networks), so much so, that the organization of the central nervous system into forebrain, midbrain, and hindbrain is thought to have originated before the protostome–deuterostome split, which is estimated to have occurred between 600 and 800 million years ago [reviewed in Ref. (27)].

The rostral to caudal organization of the *Drosophila* brain^1^ can be schematized as follows:

### The protocerebrum (forebrain)

This is the most anterior neuropil of the brain and contains many complex substructures (Figure 1B).

(1) *The Mushroom bodies (MBs)*: studies in *Drosophila* and other insects, (i.e., cockroaches and honey bees), suggest that the MBs are involved in olfactory learning and memory (LM) (28) but probably also in place memory, associative memory, context dependent sensory filtering, as well as playing a role in motor control (29–33). *Drosophila* MBs are formed by a calyx-shaped neuropil, situated in a posterior–dorsal region of the protocerebrum. The calyx then continues anteriorly into a pedunculus, which then divides into a dorsal lobe (consisting in two subdivisions, called α and α′) and a medial lobe (consisting of three subdivisions, called β, β′, and γ lobes); the β subdivision corresponds to the α subdivision of the dorsal lobe and the β′ subdivision corresponds to the dorsal lobe’s α′ subdivision (Figure 1B). At the cellular level, the MB consists of about 2500 intrinsic neurons, called Kenyon cells, which originate from globuli cells situated above the calyx.
(2) *The Central complex (CC)*: the CC is the most central and the only unpaired neuropil in the insect brain (Figure 1B). It receives multimodal inputs from most parts of the brain and has been proposed as a higher center for locomotor control, which regulates several aspects of walking and flying behavior and has also been suggested to act as a higher center for the integration of visual input as well as playing a role in spatial visual memory and place learning (34). Furthermore, there is evidence that dopaminergic neurons of the CC are involved in the control of arousal, wakefulness, and aggression [reviewed in Ref. (35)].The CC consists of four interconnected substructures: the protocerebral bridge (PB), the fan-shaped body (FB), the ellipsoid body (EB), and the noduli (N) (Figure 1B). The EB is an almost circular neuropil, lying anterior to the FB. The EB receives terminals of neurons, which originate in the protocerebrum, and it shares dendrites with parts of the FB, which lies posterior to the EB. The FB contains arborizations of terminals and dendrites linking the FB to lateral regions of the protocerebrum. The N are two nodular neuropils that receive connections from the PB neurons that also provide collaterals to the FB. The PB forms a handlebar-shaped commissural connection between the two dorsal lobes of the protocerebrum, and they also provide axons that project into the FB, from where they project further into the EB and the N.
(3) *The pars intercerebralis (PI) and pars lateralis (PL)* together with their projections to the corpora cardiaca, corpora allata, and prothoracic gland, constitute the *Drosophila* neuroendocrine system (36, 37). Most of the neurosecretory cells (NSC) are contained within the small groups of cells of the PI/PL, which are located in the dorsal medial region of the protocerebrum. The neurohemal secretory cells of the corpora cardiaca, corpora allata, and prothoracic gland form a ring-like structure partially surrounding the dorsal blood vessel. NSCs of the PI and PL secrete insulin-like peptides, FMRFamide-like peptides, pigment-dispersing hormone, corazonin, ovary ecdysteroidogenic hormone, and myomodulin (38). The corpora cardiaca/corpora allata produce juvenile hormone which, together with ecdysone, produced and released by the prothoracic gland, controls growth and molting. Recent studies demonstrated that the PI is involved in multiple behaviors and physiological phenomena, such as sleep (39, 40), locomotion (41, 42), circadian locomotor activity (43), and metabolism (44, 45), and it is considered a region functionally analogous to the mammalian hypothalamus (36).

### The deutocerebrum (midbrain)

This is the second division of the supraesophageal ganglion. The antennal lobes, which are glomerular neuropils receiving mainly olfactory receptor terminals, are part of the deutocerebrum. The deutocerebrum also receives mechanosensory input from the head surface as well as input from the optic lobes, the EB and FB of the central complex, and dendrites from ascending pathways.

### The tritocerebrum (hindbrain)

This is the third segmental preoral ganglion, which lies ventrally on either side of the gut. The tritocerebrum, similarly to the deutocerebrum, also gives rise to descending neurons and receives connections from various regions of the more anterior neuropils.

Aside from the tripartite organization of the *Drosophila* brain, recent work has drawn attention to the notion that there may be functional homology between the protocerebral neuropils and corresponding structures in the vertebrate forebrain. In particular, Strausfeld and Hirth (46) discuss evidence suggesting a deep homology of the arthropod central complex and vertebrate basal ganglia. On the other hand, Wirmer et al. (37) review the evidence suggesting the functional homology of the insect NSC of the PI/PL-copora cardiaca system and the vertebrate hypothalamus–pituitary axis. While, Farris (30) presents arguments in favor of a structural and functional homology between the MBs and the vertebrate cerebellum. What these considerations imply is that, to all intents and purposes, the fly brain can be considered a “simplified” miniature version of the vertebrate brain (47, 48). As such, it can be expected that, probably at a very basic level, the neural circuitries governing context-dependent sensory integration, LM, action and directed behavior are probably conserved from *Drosophila* to vertebrates. In fact, there is strong evidence for a conservation of the principle neurotransmitters and the associated receptor/signaling systems, with particular regard to the dopaminergic (49), serotoninergic (50), GABAergic (51), and adrenergic systems (52). In the case of adrenergic signaling, it is, however, important to point out that, although at the behavioral level, there is good evidence for a functional conservation of the so called “fight or flight” response, motivation, and aggression (in vertebrates mainly modulated by the adrenergic system); in *Drosophila*, these behaviors are instead modulated by octopamine and tyramine. Nonetheless, these two neurotransmitters are structurally and functionally related to adrenaline and noradrenaline, respectively (52), since they are all products of the metabolic transformation of the same amino acid, tyrosine. In fact, tyramine is the decarboxylation product of tyrosine, and octopamine is the β-hydroxylation product of tyramine.

## The *Drosophila melanogaster* Circadian System

### The circadian clock at the molecular level

**Figure 2:**
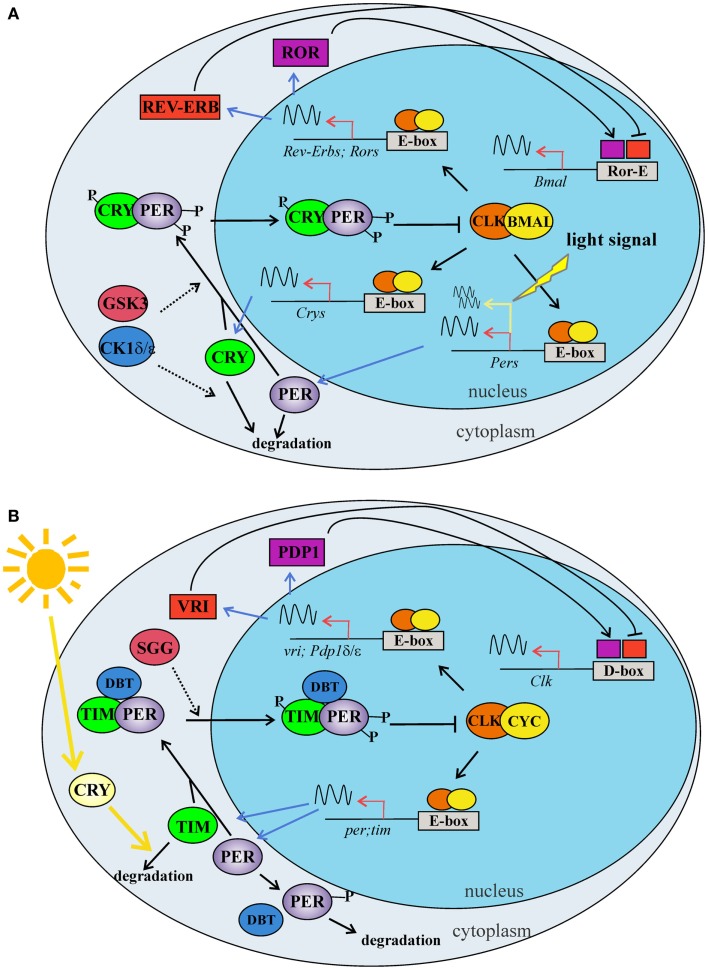
**The two major TTLs of the circadian molecular clock in mammals (A) and *Drosophila* (B)**. **(A)** The first mammalian TTL includes BMAL1 and CLK, which act as heterodimer, binding the enhancer boxes (E-boxes) in the promoter of *Per* and *Cry* clock genes. PER and CRY proteins dimerize and enter into the nucleus, where inhibit the CLK -BMAL1 activity. A second loop modulates *Bmal1* expression: CLK-BMAL1 dimers induce the transcription of *Rev-erbα* and *Ror* nuclear orphan receptor genes. REV-ERBs and RORs compete for the same element (Ror-E) in the *Bmal1* promoter, controlling *Bmal1* transcription. Phosphorylation mediated by CKs (δ/ε) and GSK3β modulate clock protein activities regulating protein–protein interactions, nuclear translocation, and degradation. Within the master clock, at the cell level, the light stimulus induces the transcription of the *Per* genes via a signal transduction cascade. **(B)** In the first TTL of *Drosophila*, CLK and CYC form a dimer, which binds the E-boxes in the promoter of *per* and *tim* clock genes. PER and TIM proteins interact in a complex, enter into the nucleus, and inhibit the CLK-CYC activity. A second TTL modulates *Clk* expression: CLK-CYC dimer induces the transcription of *vri* and *Pdp1* δ/ε genes. VRI and PDP1 δ/ε compete for the same element (D-box) in the *Clk* promoter, controlling *Clk* transcription. Phosphorylation mediated by DBT and SGG modulate clock protein activities, regulating protein–protein interactions, nuclear translocation, and degradation. In the cell, light activates the internal photoreceptor CRY, which associates with TIM and mediates its degradation. BMAL: brain and muscle ARNT-Like 1; CKδ: casein kinase; CLOCK: circadian locomotor output cycles Kaput; CRY: cryptochrome; CYC: cycle; DBT: doubletime; GSK3β: glycogen synthase kinase 3 beta; PDP1: PAR domain protein 1; PER: period; REV-ERB: nuclear receptor subfamily 1, group D; ROR: RAR-related orphan receptor; TIM: timeless; VRI: vrille; SGG: Shaggy. Dashed arrows indicate phosphorylation, while sinusoidal lines indicate transcription activity.

In *Drosophila*, as in mammals, circadian rhythms at the molecular and cellular levels are driven by interlocking autoregulatory transcriptional/translational feedback loops (TTLs). These have been recently reviewed [e.g., Ref. (53, 54)], and here we present a simplified model of the two major TTLs (Figures 2A,B). In *D. melanogaster*, the transcription factors dCLOCK (dCLK) and dCYCLE (dCYC) act as a heterodimer (dCLK/dCYC), promoting the transcription of the d*period* (d*per*) and d*timeless* (d*tim*) genes (Figure 2B). In mammals, the orthologs mCLK (or mNPAS2 in the forebrain) and mBMAL1 exert the function of positive regulators activating the transcription of the three mammalian orthologs of d*period* (m*Per1, mPer2*, and *mPer3*) and the two m*Cryptochrome* genes (m*Cry1* and m*Cry2*) (Figure 2A). In mammals, the m*Cry* genes replace d*tim* in the main TTL. Once translated, dPER and dTIM (mPERs and mCRYs in mammals) are targeted by different kinases and phosphatases, which mediate the timing of their nuclear translocation, stability, and action as negative feedback elements of dCLK/dCYC (or mCLK/mBMAL1 in mammals) regulatory activity. Among the kinases, it is worth underlining the roles played by dSHAGGY, homologous to mammalian glycogen synthase kinase-3 (mGSK3) (55), which is involved in the phosphorylation of dTIM and dPER (mPERs and mCRYs in mammals), and by dDOUBLETIME [dDBT, homologous to mammalian Casein Kinase 1 ε (CK1ε) (56)], which targets dPER (57). These factors are involved in the regulation of dPER and dTIM (mPERs and mCRYs in mammals) stability and nuclear entry and contribute to the fine-tuning of circadian rhythmicity (58, 59) (Figures 2A,B). dCLK/dCYC (mCLK/mBMAL1) are also the positive regulators of a second TTL, which (auto)controls the rhythmic expression of d*Clk* in flies and m*Bmal1* in mammals. In *Drosophila*, this TTL is under negative control by dVRILLE (dVRI), which probably competes with the positive regulator dPDP1 to bind sequence elements in the promoter region of d*Clk* (60, 61) (Figure 2B). In mammals, the second TTL is controlled by the nuclear hormone receptors mRORs and mREV-ERBs, which act as transcriptional repressors and activators of m*Bmal1*, respectively (62, 63) (Figure 2A).

In mammals, the light signal reaches the SCN via the retinohypothalamic tract (RHT) and synchronizes the master clock, promoting the transcription of the m*Per1* and m*Per2* genes, via the activation of a signal transduction cascade [reviewed in Ref. (10); Figures 1A and 2A]. On the contrary, in *Drosophila*, the ability to synchronize the clock with the 24 h environmental LD cycles is cell-autonomous and is mainly due to the light-mediated degradation of dTIM which, in turn, affects the stability of dPER. The majority of evidence concerning the molecular mechanism involved in light resetting suggests that this is mediated by the internal blue-light photoreceptor dCRY (64, 65) (Figure 2B). dCRY resets the molecular clock through a light dependent association with dTIM, which in turn activates the proteasome-mediated degradation of dTIM in a process involving the ubiquitin ligase dJETLAG (66, 67).

### The circadian clock at the organismal level

In *Drosophila*, the circadian system at the organismal level is composed by multiple oscillators located in the brain and peripheral tissues (Figure 1B). The brain master clock consists of ~150 neurons (of the ~250,000 *Drosophila* adult brain neurons), organized in bilateral pairs of clusters. In each brain hemisphere, these have been anatomically classified into 5 lateral neuron (LN) groups [including 4 large ventral lateral neurons (lLNvs), 4 small ventral lateral neurons (sLNvs), a 5th sLNv, 6 dorsal lateral neurons (LNds), and 3 lateral posterior neurons (LPNs)], and 3 clusters of dorsal neurons (DNs 1, 2, and 3) [reviewed in Ref. (68)] (Figure 1B). The anatomical subdivision does not correspond to a functional classification, and the single neurons within each cluster can show differences in protein/neuropeptide expression profiles, properties, and activities [reviewed in Ref. (68–70)]. For example, both the 4 lLNvs and the 4 sLNvs express the neuropeptide pigment dispersing factor (PDF). In the physiology of the *Drosophila* central circadian clock, PDF plays multiple roles: (i) as an output neurotransmitter, considered to be the equivalent of the mammalian circadian neuropeptide vasoactive intestinal peptide (71, 72); (ii) as a synchronizer of the oscillations of the clock neurons; (iii) playing a part in the signal transduction of light input into the circadian-neuron circuitry [reviewed in Ref. (68, 73)]. Some LNds express the long form of neuropeptide F (NPF), while sLNvs and one LNd produce the short form (sNPF). These neuropeptides are homologous to mammalian neuropeptide Y (NPY), which is involved in the control of both sleep and feeding in humans and rodents (74, 75). The role of the different neuronal clock clusters in regulating circadian activity has been investigated mainly by evaluating daily cycles of locomotor activity as a readout. In laboratory conditions (i.e., LD cycles with abrupt LD transitions), the locomotor activity of flies shows a bimodal profile, with one peak in the morning and a second peak in the evening. Several studies indicated that these two peaks are governed by specific morning-(M) and evening-(E) coupled clocks, which have been located to the 4 sLNvs (M), and in the 5th sLNv, the LNds, and the DNs (E), (76, 77). While initial studies suggested a dominant role of the M clock in the control of the endogenous rhythm (78), recent data indicate that the network of all circadian clock neurons contributes to the generation of behavioral rhythmicity, in both constant darkness (DD) and LD conditions (79, 80).

Recent work has begun to clarify how the master clock communicates with other brain regions to give rise to circadian rhythmic locomotor behavior (43). It has, in fact, been demonstrated that the time-of-day information generated by the circadian clock network of the sLNvs, and probably the LNds, is sent to the DN1s, which in turn contact specific neurons (identified by the driver *Kurs58Gal4*) of the PI (Figure 1B). The *Kurs58* neurons probably consist of at least two different neuronal populations, which seem to have opposite roles in the control of the sleep/wake rhythm, and their relative contribution might vary during the 24 h (43). Interestingly, Cavanaugh and colleagues identified DH44, the *Drosophila* homolog of the mammalian stress hormone Corticotropin releasing factor, as a possible candidate signaling molecule important in the PI-modulation of locomotor activity rhythms. In addition, since DH44 receptors have been identified in a group of cells of the lateral protocerebrum, probably involved in stress-induced locomotor activity (81), the authors suggest a possible parallelism with mammals in which the stress glucocorticoids released from the HPA axis show a circadian production and act as synchronizing signals for the peripheral and possibly also for the central clocks (43, 82).

In *Drosophila*, in addition to the clock neurons, glial cells (in particular astrocytes) contribute to the daily control of locomotor activity rhythms (83, 84). In this activity, *ebony*, which encodes for an enzyme involved in dopamine and histamine recycling, seems to play a key role as an output gene (83). Moreover, glia might act by modulating PDF transport and/or release from vLN projections, indicating the importance of a glia-to-neuron communication in the control of behavioral rhythmicity (84). Interestingly, a role for the glial cells has been suggested also for circadian behavior in mammals (85).

In contrast to mammals, in which virtually every area of the brain possesses a functioning peripheral clock (Figure 1A), in *Drosophila*, brain regions outside the master clock network and glia apparently do not contain all the molecular elements necessary for a functional clock. However, a study performed in 2000 revealed that several independent d*per*-promoter- and d*tim*-promoter-*Gal4* lines (tools commonly used in *Drosophila* circadian studies) showed activity in non-master clock brain neurons and structures. Among them, the PI, the EB, and the FB were labeled in the *perGal4* strains, while neurons located dorsolaterally to the antennal lobe, near the tritocerebrum, and the subesophageal ganglion were marked in the *timGal4* lines. Such neurons are normally not (or weakly) detected with anti-dPER or anti-dTIM antibodies (86). In 2008, Yoshii and colleagues demonstrated the presence of dCRY in about 12 neurons per brain hemisphere, which resemble the R4 EB neurons of the central complex (87). Moreover, recently, dPER and dCRY proteins were detected in two neurons located in the dorsal-anterior-lateral (DAL) protocerebrum and implicated in a form of long-term memory (88).

As in mammals, non-central-brain peripheral circadian clocks are present in multiple districts of the *Drosophila* body, such as the compound eyes, antennae (89, 90), gustatory neurons (91), prothoracic gland (92), Malpighian tubules (93), and oenocytes (94) (Figure 1B). In the simplified model of the mammalian circadian system, the SCN master clock controls the phase of all the peripheral clocks, with the exception of the semi-autonomous oscillator in the olfactory bulb (95). This organization is not completely transposable to *Drosophila*. Experimental evidence instead suggests that in *Drosophila*, some peripheral clocks, such as those of the antenna (which control circadian odor-sensitivity), those of the proboscis (controlling the gustatory physiology rhythms), and those of the Malpighian tubules (the renal organ of the fly), are autonomous systems which might oscillate in phase with the master clock by directly perceiving and responding to the same environmental stimuli (91, 96, 97). However, there are at least two exceptions: it has been demonstrated that the central clock controls both the non-autonomous clock in the prothoracic gland, which regulates the rhythm of pupal eclosion (92), and the phase of clock timing in the oenocytes (which are involved in the synthesis of sex pheromones secreted on the cuticle surface), via PDF signaling (98). Interestingly, the pheromones synthesized by the oenocytes are not only involved in mating (98) but, to a lesser extent, also in male aggressiveness (99).

## Circadian Clock, Sleep, Learning and Memory, and Social Interactions

In humans and vertebrate models, complex behaviors such as sleep, as well as LM, appear to be influenced in some way by the circadian system or genes, even if the mechanistic relationship is still unclear. Both sleep and LM are often impaired in psychiatric patients. In particular, the majority of patients with schizophrenia, bipolar, and depressive disorders refer sleep disturbances [insomnia or sleepiness; reviewed in Ref. (100, 101)]. Neuropsychiatric patients may also be characterized by impairments in the selection of positive or negative information from working memory and in their capability to access episodic memories (102). In addition, many psychopathologies may display impairments in interpersonal interactions (103). As illustrated in the following sections, several data suggest an involvement of the circadian clock or genes in the determination of these complex behaviors also in *Drosophila*.

### Sleep

Sleep is controlled by both circadian and homeostatic systems. In the mammalian brain, the core sleep circuit is formed by interconnected sleep and arousal centers, which include hyptothalamic GABAergic sleep-promoting and orexin-positive wake-promoting areas, which project to multiple brain regions [reviewed in Ref. (104, 105)] (Figure 1A). Recently, glial astrocytes have been implicated in the regulation of sleep homeostasis (106). In mammals, several lines of evidence indicate that the circadian clock controls the timing of the sleep-wake cycle. For example, in humans, mutations, which alter the phosphorylation site of PER2 or of the CKs, have a causal role in circadian-based sleep disorders such as the advanced and delayed sleep phase syndromes [reviewed in Ref. (24)]. In addition, studies on mammalian models suggest that mutations at the level of some circadian clock genes affect sleep homeostasis. For example, *Clk* mutant mice sleep on average 2 h less than wild-type individuals (107), while both *Bmal1* knock-out (KO) and *Cry1/Cry2* double KO mice show an increase in their total sleep time (108, 109). However, this is not a general rule for circadian clock genes since both single or double gene mutants for m*Per1* and/or m*Per2* do not show abnormalities in sleep homeostasis (110). These data seem to suggest a pleiotropic non-circadian effect of some of the circadian genes in the control of sleep homeostasis.

More than a decade ago, it was shown that *D. melanogaster* has a sleep-like condition [reviewed in Ref. (111)] associated to a decrement in both sensory responsiveness and brain activity (112, 113). Behaviorally, each bout of the fly sleep-like state is defined as a period of inactivity lasting ≥5 min, and day or night time sleep are calculated by summing up the total bouts of sleep time occurring in the light or dark period, respectively (114, 115). Using this behavioral parameter and the powerful transgenic toolbox available for this insect, it has been demonstrated that, as in humans, in fruit flies GABAergic neurotransmission and serotoninergic signaling promote sleep (116, 117), while dopaminergic neurons stimulate arousal and wakefulness (118, 119). Differently to humans, however, *Drosophila* does not seem to have an orexin-based wake-promoting neurotransmitter system (120), which might be substituted instead by PDF, together with octopamine (40, 121). Nonetheless, recent work has extended the parallelism between mammalian and *Drosophila* sleep physiology demonstrating, via both behavioral and electrophysiological recordings, that also in flies sleep intensity varies during the 24 h (122). In particular, deeper sleep phases occur mainly during the night and have been hypothesized as being associated to a synaptic downscaling process, similar to that proposed for mammals (122). It is noteworthy that the presence of similarities between mammalian and *Drosophila* sleep allowed the design of a strategy based on the analysis of the sleep-like state in genetically modified or pharmacologically treated flies, which then led to the identification of amylase as a possible biomarker of sleepiness in humans (123).

As in mammals, *Drosophila* sleep is subject to both circadian and homeostatic regulation (124, 125). It has also been shown that some clock genes, such as d*cycle* and d*Clk*, might be involved in sleep homeostasis, while others such as d*per* appear not to have a role in the control of this phenomenon (124, 126, 127), again suggesting a non-circadian role for those circadian genes, which do affect sleep homeostasis. Brain areas involved in fly sleep regulation include the PI, which harbors both *Dilp2*-positive wake-promoting and EGFR ligand-expressing sleep-promoting neurons (39, 40), the sleep-promoting regions of the MBs (128, 129), and the FB of the central complex (130, 131) (Figure 1B). In addition, part of the circadian clock network is important in sleep regulation, with opposing effects. It has been demonstrated that 4 sLNvs promote sleep during the night, while the lLNv neurons have a role in wakefulness and arousal (132–136) (Figure 1B). In particular, the sleep promoting role of the sLNv neurons seems to be mediated by the neuropeptide sNPF, which likely acts with other neuromodulators in multiple brain areas involved in the sleep/wake state (136). The neuropeptide involved in the wake promoting role of the lLNvs is PDF, which probably has an effect on sLNvs and other brain regions expressing the PDF receptor, such as those involved in locomotion control (i.e., the EB) (132). In addition, lLNvs express the sNPF receptor, probably important for the sLNv–lLNv coordination (136). Moreover lLNvs produce the *Rdl* GABA_A_ and GABA_B_-R2 receptors, which are a likely target of the GABAergic sleep-promoting neurons (132, 133, 137). Interestingly, hyperexcitation of the LNv leads to a more fragmented nocturnal sleep of flies (133). Finally, several lines of evidence indicate that the role of LNvs in sleep regulation and homeostasis is independent from their role as circadian clock neurons, since genetic manipulations of LNvs which alter sleep homeostasis do not modify their circadian functions, evaluated both as circadian locomotor activity or circadian expression of molecular clock components (133, 136, 137).

### Learning and memory

In healthy individuals, human cognitive processes are tightly connected to the sleep/wake cycle, and during the 24 h day they show a progressive deterioration associated to the increased amount of time spent awake (138). However, the decrement in cognitive performance is not linear with increasing sleep pressure, and several lines of evidence indicate the impact of circadian regulation in cognition, with circadian variations in the ability to learn as well as memory acquisition and retrieval (139–142).

In mammals, the hippocampus (HP) plays an important role in learning processes, formation of new memories, as well as in consolidation from short- to long-term memories (Figure 1A). Similarly to the majority of the brain regions in mammals, the HP expresses clock genes in a rhythmic manner (143). In addition, the HP possibly receives both direct and indirect circadian input from the SCN and other peripheral oscillators [reviewed in Ref. (141)]. Mice mutants for many circadian clock genes (such as m*Bmal1*, m*Cry1*, m*Cry2* and m*Npas2*) show impairments in different types of LM tests (144–146). Although such data do not exclude *per se* non-circadian pleiotropic effects of these mutations, nevertheless, the importance of a functional circadian system in hippocampal-dependent LM has been suggested from studies in humans which indicate that both shift-work and chronic jetlag cause cognitive impairments (16, 147). Similar indications have been obtained both following manipulation of the environmental LD cycle and in SCN-lesioned animal models [reviewed in Ref. (141)]. However, the importance of the SCN in these processes remains controversial (141).

Hippocampus-dependent LM has been associated to several biological processes and phenomena, which rhythmically occur in this structure. For example, the formation and persistence of long-term memory has been associated to the signal transduction pathway, involving the cyclic adenosine monophosphate (cAMP)/cAMP response element binding protein (mCREB) and mitogen activated protein kinase (mMAPK) (148–150). In the HP, cAMP/mCREB and mMAPK are rhythmically expressed (150, 151) and are also part of the molecular clock (152–155). For example, the phosphorylation of mBMAL1 by mMAPK inhibits the mBMAL1/mCLK-dependent transcription of the m*Per* and m*Cry* genes (156). The m*Per1* and m*Per2* promoters contain cAMP responsive element (CRE) sites that bind mCREB in order to enhance their transcription (157). It has been demonstrated that the disruption of mMAPK oscillations interferes with the persistence of long-term memory (158), and both m*Per1* and m*Per2* KOs show defects in hippocampal-dependent learning tasks and reduced levels of phosphorylated mCREB have been demonstrated in m*Per2* KOs [reviewed in Ref. (141)]. Other phenomena linked to LM processes, which show daily rhythmicities, are synaptic morphology (159) and adult neurogenesis (160). In particular, there are several indications concerning the circadian regulation of both phenomena. About 24 h rhythms in neuronal proliferation and changes in dendritic complexity and spine density have been observed in several rodent species (161–165). Circadian disruption caused by phase shifts negatively impacts both neurogenesis and synaptic complexity and can reduce the performance in hippocampal-dependent memory tasks in several species [reviewed in Ref. (141)]. Moreover, adult neurogenesis is impaired in mice harboring mutations in different circadian clock genes, such as m*Per2* and m*Rev-erb* α (166).

*Drosophila melanogaster* shows several types of LM, such as olfactory LM, courtship conditioning LM, spatial and visual LM [reviewed in Ref. (167, 168)]. Most of these memories are characterized by different phases: short (minutes), middle (hours), and long, which can persist for several days (169, 170). The possible involvement of the circadian clock in fly LM performance has been mainly evaluated using the olfactory and courtship conditioning paradigms. In the olfactory LM assay, flies learn to associate conditioned stimuli (CS i.e., an odorant stimulus) to an unconditioned stimulus (US i.e., an electrical shock) (171), while in the courtship conditioning paradigm attractive pheromones act as the CS, while aversive pheromones act as the US (172). The MBs are the key structures for olfactory LM, with the γ lobes mainly required for short-term memory (STM) formation, and α/β neurons involved in the establishment of long-term memories (LTMs) (28, 173) (Figure 1B). In addition to MBs, EB also appear to contribute to LTM (174) (Figure 1B). In the courtship conditioning paradigm, the antennal lobes (and perhaps also the optic lobes) seem to be particularly important in the formation of the first part of memory (up to 30 min), while the MBs have been implicated in the subsequent phases (from 30 min to several days) (175). As in mammals, the cAMP signaling pathway plays a central role in *Drosophila* memory and, while STM involves cAMP-dependent modifications of existing proteins [e.g., ion channel activities, either directly or indirectly via phosphorylation by PKA (170, 176)], LTM requires cAMP/CREB-mediated transcription (177).

Several lines of evidence suggest that the circadian clock impacts olfactory LM in flies. Under 12:12 LD cycles, *Drosophila* shows a 24 h rhythm in learning efficiency, which has been hypothesized to be associated with the rhythmic variation in abundance of the *Drosophila* mCREB homolog, dCREB2, in adult heads (178). Interestingly, dCREB2 appears to be under circadian control in several brain regions, including the MBs (179), and plays a crucial role in circadian rhythmicity, since mutations in dCREB2 alter circadian locomotor activity probably by modifying the transcriptional oscillations of the d*per* gene (180). In addition, it has been demonstrated that in wild-type flies, olfactory STM is under circadian control, with a peak of memory performance at the beginning of the night, both in LD and in DD conditions (181). The STM rhythmicity was lost in LL conditions and in *per*^0^ and *tim*^0^ mutant flies tested in DD. Since *cry*^b^ mutants, which possess a non-functional peripheral oscillator in the antenna, maintain STM rhythmicity in DD, the authors suggest that the circadian STM modulation is due to the central oscillator, which might govern the availability of molecules involved in the memory-based signal transduction cascade (181). The circadian clock gene d*per* has also been implicated in *Drosophila* LTM, in both the olfactory learning and courtship conditioning paradigms (88, 182, 183). The role of d*per* in olfactory and courtship LTMs appeared to be independent from the circadian clock, since other clock mutants, such as *tim*^0^, *Clk-Jrk*, and *cyc*^0^, showed normal LTM (88, 182). Although the protocol differences in the two LM paradigms complicate the direct comparison of the results, it is interesting to note that d*per* is required outside the MBs in both cases. In particular, the *per*-dependent control of olfactory LTM maps at the level of the DAL neurons in the dorsal lateral protocerebrum (88), while for the courtship LTM the presence of d*per* is required at the level of the FB of the central complex (88, 183). The activity of these neuronal structures seems to be mainly important for the LTM recall, rather than LTM formation and storage (88, 183). In addition, both types of memory required dCREB2 activity, although the mechanistic link with d*per* has not yet been explored (88, 183).

### Social interactions

A suite of social behaviors, including courtship, aggression, mating, the recognition of conspecifics, have been identified in flies [reviewed in Ref. (184)]. In *Drosophila*, several lines of evidence suggest a link between social behavior and the circadian clock. For example, behaviors such as courtship and mating show a circadian timing (185–188). In addition, an interaction between social behavior and the circadian clock has been demonstrated by Levine et al. (189), who showed that the phase of circadian locomotor activity is modulated by social context. In fact, the locomotor activity phase of wild-type flies was more dispersed in the presence of *per*^0^ (arrhythmic) mutant flies and advanced in the presence of *per*^Short^ (*per*^S^) individuals, which are characterized by a circadian clock with a short periodicity (190). These effects were found to be dependent on many variables, such as the size and relative genetic composition of the group, the time of the day, and the ability to perceive odors, since anosmic flies were insensitive to the modification of the social context (189).

Following evaluation of the relationship between mating behavior and the circadian clock, it was shown that the production of sex pheromones in males, which occurs at the level of specific cuticular cells named oenocytes, is clock-regulated and controlled by a peripheral circadian clock within the oenocytes themselves (94). The oenocyte molecular clock appears to be less autonomous with respect to those of the other peripheral tissues, since the master clock is able to modulate the phase of oenocytes via PDF, which might act as a neuroendocrine signal (98). In flies, social context can act as an input signal to the clock (zeitgeber), influencing the expression of circadian clock genes at the level of the central nervous system as well as in the periphery (94, 98), thus modulating the circadian locomotor behavior (189), as well as other behavioral outputs such as mating and possibly other aspects of social behavior. For instance, it is interesting to note that oenocytes are also important in the regulation of male–male social interactions, since mutant males lacking oenocytes show low levels of aggression and high levels of male–male courtship compared to wild-type flies (99).

## The Study of the Link between Circadian Clock and Neuropsychiatric Diseases in Flies

To our knowledge, the only studies conducted in *Drosophila*, which could be thought of as addressing the link between the circadian clock and neuropsychiatric diseases, were performed independently by two different research groups some years ago (191, 192). Both analyses evaluated the effects of the drugs, lithium and valproate (192), or lithium only (191), on *Drosophila* circadian locomotor activity.

Lithium and valproate are mood stabilizers that have been widely used for the treatment of bipolar disorder (193–195). To a more limited degree, valproate is also used in the treatment of schizophrenia (196). Although the mechanisms of action of lithium and valproate are still not completely understood, it is known that both types of drugs affect several biological phenomena which might be related to their therapeutic effects, including neurotransmitter release, monoamine metabolism, neuronal excitability, adult neurogenesis, as well as different circadian parameters (197–201). These effects are possibly the result of a direct inhibitory activity of both drugs on the G-proteins, myo-inositol monophosphatase (mIMP), and the mGSK-3α and mGSK-3β isoforms (197, 200).

Similarly to mammals, in *Drosophila*, adults chronic administration of lithium, within doses normally employed to treat human mood disorders, determines an increase in the periodicity of free-running circadian locomotor activity rhythms (191, 192). Analogous results were obtained following valproate treatments, although the effect was weaker with respect to that observed in the case of lithium; in particular, valproate was also more toxic to the flies (190). These reports further suggest that lithium possibly affects fly circadian periodicity by acting on dSHAGGY, the *Drosophila* ortholog of mGSK-3β (Figures 2A,B). As mentioned earlier, this kinase is part of the molecular clockwork and acts by phosphorylating dPER and dTIM, thereby regulating their daily nuclear translocation which contributes to the fine tuning of circadian rhythmicity (58, 59). Dokucu and colleagues showed that flies heterozygous for a null *shaggy* mutation as well as individuals overexpressing d*shaggy*, specifically in *pdf*-expressing neurons, showed a lengthening of their circadian periodicity after chronic exposure to lithium (190). It is worth underlining that heterozygote *shaggy* null mutants are characterized by longer circadian periodicity compared to controls, while overexpression of d*shaggy* in *pdf*-neurons leads to short-period circadian locomotor activity. Furthermore, Padiath and colleagues showed that lithium-treated flies were characterized by a reduced activity of the fly mGSK-3β homolog (189).

Even though the above studies evaluated a single behavioral trait, these data still suggest that *Drosophila* might represent a useful translational animal model to screen for candidate drugs suitable for the treatment of neuropsychiatric disorders. In addition, the similarity between the effects obtained on the circadian parameters and the molecular target/s in both mammals and flies supports the idea that *Drosophila* might be used in the evaluation of the link between the circadian clock and neuropsychiatric diseases.

## The “Omic” Approach to *Drosophila* Behavioral Analyses

The multifactorial nature of mental illnesses makes the study of neuropsychiatric diseases in animal models extremely difficult. One of the major problems arises from the lack of specific biomarkers for the different types of these disorders. In addition, psychiatric patients are characterized by several behavioral traits and it is difficult to have reliable tests, which mimic psychiatric-like behaviors in non-human organisms. On the other hand, specifically in the case of flies, it is practically feasible to subject experimental groups of animals to batteries of behavioral tests, the main characteristics of which are described below. The value of these assays does not lie so much in the information that each can provide singularly but, in keeping with the complex behavioral aspects typical of neuropsychiatric illnesses, in the information that the whole set can provide collectively.

*Susceptibility of flies to seizures* can be evaluated following hyperstimulation by mechanical shock (202). Following mechanical hyperstimulation, modifications of the original protocol allow flies to be assayed so that following the mechanical shock, flies are not only assayed for the time taken to recover from the temporary seizure, but they are also video-recorded in order to evaluate the time taken to climb to different heights of the vial in which they are contained (203). The assay performed in this way allows not only the determination of the post-hyperstimulation recovery of flies from seizure, but also the time taken to regain locomotor coordination.

*Nociception* can be determined using a heat-plate test paradigm [i.e., as described in Ref. (204)]. In this paradigm, avoidance of noxious heat is determined by placing groups of flies in a sealed experimental chamber, in the dark. The bottom of one end of the chamber is heated to 46°C using a computer controlled-thermoelectric (Peltier) element, and following a period of 4 min the distribution of flies within the chamber and the percentage of avoidance are estimated based on counting the number of flies that fail to avoid the noxious temperature, compared to the total number of flies in the chamber.

Recent advances in the field of machine vision and video-tracking techniques have led to the development of an open source software project called Ctrax (205). Continuous high definition filming and tracking of up to 20 flies moving simultaneously in a single arena for periods of up to 1 h is a realistic experimental condition. The tracking data are then subjected to analysis to provide (i) qualitative and quantitive data on *social interactions* occurring between pairs of flies during the whole tracking period; (ii) basic locomotor information from the single flies, such as distance traveled, speed of locomotion, turning angle, and time spent moving. From the latter, it is also possible to extrapolate information regarding cognitive aspects of locomotion, which relate to *decision making processes* active during the exploration of the arena by the individual flies.

*Optokinetic response and attention* can be determined by using a “maze” approach as described in Ref. (206). This apparently simple paradigm, in its basic form, provides a measure of the integrity of the neuronal circuitry underlying the perception and elaboration of visual information. Through manipulation of the experimental conditions, it is also possible to evaluate the integrity of attention-like processes.

*Circadian analysis* can be conducted as described in detail in (115, 205, 207). Whichever approach is adopted, the analysis of *sleep* patterns, based on the criteria originally defined by Shaw et al. (125), can be performed using the open source PySolo software package, as mentioned above (114, 125).

The determination of *food preference* in groups of flies can be established using the two-taste discrimination test (208). The test allows the evaluation of two kinds of output. (i) On the one hand it is possible to precisely quantify the preference between attractively tasting and unpleasantly flavored feeding solutions. (ii) The second possibility is to determine the tendency of flies under certain conditions (i.e., genetically, pharmacologically and/or environmentally determined) to prefer feeding solutions laced with known quantities of addictive compounds, such as ethanol, with respect to the standard feeding solution (209). The latter modality of conducting the test allows the evaluation of addiction/reward-seeking behavior of flies.

A rather recent approach consists in a learning paradigm, which the authors (210) called *no-idleness learning*. The response (or rather, lack of response) of an organism to unrelentingly uncontrollable noxious environmental variations is governed by a special kind of LM called learned uncontrollability. Under such conditions, an animal can learn that there is no patterned behavioral response which can be used to avoid the noxious effects of the essentially unpredictable environmental variations. In particular, in *Drosophila*, learned helplessness appears to consist in a cognitive element, which is the learned uncontrollability, and a motivational component, which consists in the decreased behavioral activity of the animal under the stressful conditions (209).

## Discussion and Future Perspectives

*Drosophila* has been recently proposed as a model organism to study neuropsychiatric disorders (211–213). The main reasons which justify this choice are that: (i) flies show a relatively simple brain, which shows interesting functional homologies with important regions of the mammalian brain; (ii) the fundamental neurobiological processes and neurotransmitter systems are conserved; (iii) flies are characterized by a set of complex behaviors such as sleep/wake cycles, LM and social interactions, which can be modulated by experience. Similar endophenotypes are often impaired in neuropsychiatric patients. *Drosophila*, therefore, represents a powerful tool for the comprehension of the molecular, cellular, and neural circuit mechanisms, which form the basis of such behaviors in both normal and impaired conditions (211).

In the study of the possible link between psychiatric diseases and the circadian clock, the use of *Drosophila* might provide an advantage for three main reasons: (i) the *Drosophila* circadian system organization is simpler compared to that of mammals; (ii) genetic mutations at the level of circadian clock genes, which cause modifications in the timing of the circadian clock, are available such as, for example, the two d*per* gene mutants, which show shortening (*per*^S^) or lengthening (*per*^Long^, *per*^L^) of the clock periodicity (190); (iii) *Drosophila* has homologs to most of the candidate genes associated with psychiatric diseases, as reported in case-control and genome-wide associations (GWA) studies (212, 214, 215). For these genes, *Drosophila* null or knock-down mutants are already available or easily obtainable (i.e., from the public fly mutant repositories). It is, for example, noteworthy to mention d*Dmca1D*, the fly homolog of m*CACNA1C* (or m*CaV1.2*), a gene encoding for an L-Type voltage-gated calcium channel which has been found in several GWA analyses to be associated with both schizophrenia and bipolar disorders (216, 217). Interestingly, it was demonstrated that in mammals the expression of mCav1.2 is rhythmic and modulated by the circadian clock element mREV-ERBα. In addition, mCav1.2 appears to be involved in resetting of the circadian clock by light (218). In *Drosophila*, it is relatively easy to construct strains in which null mutations in psychiatric-disease-candidate genes are carried in a “sensitized” genetic background (i.e., having a slightly perturbed circadian clock, as in the case of the *per*^S^ and *per*^L^ mutants). The double or single mutants can then be tested in stress-inducing environments, which might be represented, for example, by extreme LD cycles or a condition of sexual deprivation (209). This approach would open the possibility of evaluating both Gene X Environment (G X E) and Gene X Gene X Environment (G X G X E) interactions, which probably constitute the basis of the multifactorial nature of psychiatric diseases.

In *Drosophila*, experimental designs exploring G X E and G X G X E interactions could be used to test the hypotheses which link the circadian clock to psychiatric disorders in humans. For example, it should be possible to evaluate the idea that an increased risk for psychiatric disease is associated with an environmentally and/or genetically induced misalignment between the central and the peripheral clocks. One of the possibilities to approach this problem in *Drosophila* could be to analyze the effects of such perturbations on parameters of social interaction and their underlying neuronal circuitries. In fact, as illustrated in the previous sections, the relationship between the master clock and the peripheral oenocyte clocks, which have been shown to play an important role in modulating fly social interactions, resembles the situation in mammals in which the autonomous central clock governs the phase of “slave” peripheral clocks. Moreover, the hypothesis of a direct influence of the master clock on psychiatric disease might be evaluated using the behavioral outputs of the learning and short-term memory phenotypes, which are modulated by the clock both in *Drosophila* and mammals. However, differently from mammals, the *Drosophila* brain regions involved in the control of these phenotypes apparently do not contain a running clock, but are directly or indirectly influenced by the master clock. Therefore, this represents a simplified system to evaluate G X E and G X G X E interactions. Finally, another possibility is to conduct the G X E and G X G X E analyses using the “omic” approach, i.e., by evaluating several relatively simple traits in parallel in a “behavioromic” type of approach. With the *Drosophila* powerful transgenic toolbox, it should then be possible to identify the specific neuronal circuitry governing the selected behaviors and to characterize the activity of such neuronal circuits during development and adulthood as well as in response to pharmacological treatments.

As has already transpired from this, as well as from other excellent reviews, it is clear that in *Drosophila*, given a behavioral paradigm it is straightforward to choose a genetic approach in order to define the molecular, cellular, and circuit mechanisms, as well as the pathogenesis of impairments caused by specific genetic, pharmacological, and/or environmental manipulations. In particular, *Drosophila* is a choice model organism in which to test the *in vivo* therapeutic potential of large numbers of chemicals as a first tier approach in the translation to mammalian models and humans. In this respect, the possibility of testing relevant behavioral end points in a high throughput manner is of particular importance. Currently, high throughput designs have been described for the evaluation of: (i) circadian patterns of activity/sleep (115); (ii) optomotor performance and attention (219); (iii) locomotor activity and social interactions following simultaneous tracking of multiple flies (205). In conclusion, we argue that the molecular/genetic, cellular, and neurobiological features of *Drosophila* make this a choice translational model in which to test the causal link between genetic and/or environmental manipulations leading to perturbations of the circadian system and defects in a suite of behavioral traits which, collectively, would address some of the key neurological features involved in neuropsychiatric diseases. Such investigations could prove of extreme value in pointing the way for more focused studies in model organisms evolutionarily closer to humans.

## Conflict of Interest Statement

The authors declare that the research was conducted in the absence of any commercial or financial relationships that could be construed as a potential conflict of interest.

## Footnotes

^1^We wish to acknowledge the Flybrain web site (http://www.flybrain.org) for detailed information on the *Drosophila* brain structure and function.
